# Morphometric and Cellular Evaluation of Delaminated Cartilage From the Acetabulum

**DOI:** 10.1177/23259671261450542

**Published:** 2026-07-21

**Authors:** Sebastian Gebhardt, Janosch Schoon, Emely Bortel, Georgi I. Wassilew, Alexander Zimmerer

**Affiliations:** † Center for Orthopaedics, Trauma Surgery and Rehabilitation Medicine, University Medicine Greifswald, Greifswald, Germany; ‡ Xploraytion GmbH, Berlin, Germany; § Gelenkquartier, ORTHOPÄDIE & UNFALLCHIRURGIE, Karlsruhe, Germany; Investigation performed at the Center for Orthopaedics, Trauma Surgery and Rehabilitation Medicine, University Medicine Greifswald, Greifswald, Germany

**Keywords:** hip arthroscopy, cell biology, platelet-rich plasma

## Abstract

**Background:**

Delaminated acetabular cartilage flaps are associated with inferior outcomes of arthroscopic treatment of femoroacetabular impingement syndrome. Recently, concomitant arthroscopic minced cartilage implantation has shown encouraging early clinical results under such circumstances.

**Purpose/Hypothesis:**

The purpose of this study is to investigate the morphology, outgrowth, and functionality of chondrocytes from delaminated acetabular cartilage flaps in comparison to controls, in order to assess their suitability for minced cartilage implantation. It was hypothesized that despite morphologic differences, chondrocyte viability and functionality do not differ between groups.

**Study Design:**

Controlled laboratory study.

**Methods:**

Delaminated acetabular chondral flaps were obtained from 11 patients undergoing hip arthroscopy. Control samples were taken either from the acetabulum in patients undergoing total hip arthroplasty for avascular necrosis (2 samples) or from patients undergoing minced cartilage implantation at the knee (12 samples). Synchrotron radiation microcomputed tomography (SR-µCT) analyses and histologic evaluations were conducted. In vitro investigations assessed cell proliferation, metabolic activity, and redifferentiation potential in 3-dimensional spheroid cultures.

**Results:**

Microcomputed tomography revealed a significantly increased mean number of chondrocyte lacunae in delaminated acetabular cartilage compared to control samples, along with a significant reduction of lacunar diameter. Morphometric and histologic analyses demonstrate cell-free, necrotic areas with fiber structures within delaminated acetabular cartilage samples. Regarding the number of outgrown cells, metabolic activity, and proliferation capacity, no differences between delaminated acetabular cartilage and control samples were observed. Initially cultured chondrogenic spheroids of cells from delaminated acetabular cartilage samples had lower protein and proteoglycan content compared to controls, but no differences were observed for spheroids cultured with TGF-β1 supplementation.

**Conclusion:**

Delaminated acetabular cartilage exhibits altered morphology. However, cell numbers, outgrowth potential, and population doublings of chondrocytes isolated from delaminated acetabular cartilage were comparable to those of controls. Redifferentiation of chondrocytes isolated from delaminated acetabular cartilage relies on TGF-β1 stimulation.

**Clinical Relevance:**

Delaminated acetabular flaps are strongly degenerated, characterized by atrophic chondrocyte lacunae and tissue necrosis, indicating that autograft utilization is unfavorable. However, cell outgrowth and functionality suggest that delaminated acetabular cartilage flaps provide a suitable tissue source for minced cartilage implantation after augmentation with TGF-β1.

Delaminated acetabular cartilage flaps are frequently observed during arthroscopy for the treatment of femoroacetabular impingement syndrome (FAIS).^6,20,23^ This type of cartilage lesion is associated with an inferior outcome following arthroscopic treatment of FAIS.^29^ Commonly, delaminated cartilage flaps are debrided, and the exposed subchondral bone is treated by microfracturing (MFX).^8^ However, for this procedure, complications such as subchondral bone cyst formation and unsatisfactory outcomes have been reported.^12^ The feasibility of reattaching delaminated acetabular cartilage flaps by fibrin glue or suture repair has also been investigated. In this regard, the viability of chondrocytes isolated from delaminated acetabular chondral was reported from 39% to 90%,^14,19,22,24,33^ and beneficial clinical short-term effects of direct reattachment techniques were reported.^28,30,31^ However, histologic analyses of tissue structure of delaminated acetabular chondral flaps were shown to be severely different from healthy cartilage,^14,19,24^ and a high reoperation rate with conversion to total hip arthroplasty (THA) was observed for direct reattachment techniques at mid- and long-term follow-up.^21^ Cell-based cartilage repair by autologous cartilage implantation (ACI) has been reported with good results,^18^ and cartilage tissue from delaminated acetabular flaps was shown to be an adequate source for donor cartilage tissue.^5^ However, ACI is a 2-stage procedure, associated with regulatory obstacles and high treatment costs, circumstances that restrict its widespread availability.^2^ An alternative cell-based cartilage repair technique, the all autologous, arthroscopic minced cartilage implantation (MCI), has recently shown encouraging clinical results for the treatment of acetabular cartilage lesions.^9^ While a recent in vitro investigation of cartilage tissue harvested during arthroscopic MCI has shown adequate cell outgrowth and function, significant interindividual differences between cartilage tissue derived from different donors were observed, which implies that the quality of the donor side cartilage tissue may be crucial for the results of MCI.^10^ In this regard, it is of utmost importance to investigate whether cartilage tissue harvested from delaminated acetabular chondral flap tissue can serve as an adequate tissue source for MCI during hip arthroscopy.

In the present study, we investigated the morphologic structure of acetabular cartilage flaps using 3-dimensional (3D) microcomputed tomography based on synchrotron radiation (SR-µCT) compared with intact acetabular cartilage harvested during a THA for avascular necrosis. We furthermore compared chondrocyte outgrowth, viability, proliferation, and chondrogenic redifferentiation potential with control samples derived from knee joints during MCI. Comparison to samples originating from the knee seemed appropriate, as recent clinical studies have demonstrated favorable clinical and radiologic outcomes of MCI in the knee at short- and mid-term follow-up.^3,4,26,27^ It was hypothesized that despite morphologic differences, chondrocyte viability and functionality do not differ between groups.

## Methods

### Patient Recruitment and Sample Harvest

Tissue samples were obtained from 11 consecutive patients diagnosed with FAIS and delaminated acetabular chondral flap lesion (FLAP) undergoing hip arthroscopy. The FLAP samples obtained during hip arthroscopy were resected in total, and the resulting defect was treated by collagen membrane augmented bone marrow stimulation. Twelve control cartilage samples were taken by curettage from healthy cartilage at the border of cartilage defects at the knee joint, which is a necessary preparation in conjunction with cartilage repair (CR) surgeries. Further, 2 cartilage samples were taken from intact lateral acetabular cartilage of a patient undergoing THA due to avascular necrosis (CR-a) (Table 1). All samples were collected intraoperatively, immediately placed in sterile phosphate-buffered saline (PBS), and transferred to the laboratory within the same clinical facility after surgery. This ensured consistent handling and minimized variability between specimens. Ethics approval was obtained from the local ethics committee at University Medicine Greifswald in accordance with the World Medical Association Declaration of Helsinki. All patients provided written informed consent.

**Table 1:** Patient Demographics and Sample Data*^*a*^*

Patient No.	Group	Patient Age, y	Sex	Indication for Surgery	ALAD Classification	Sample Weight, g
1	FLAP 1	32	M	FAIS	3	57.3
2	FLAP 2	28	M	FAIS	3	135.5
3	FLAP 3	32	M	FAIS	3	121.3
4	FLAP 4	25	M	FAIS	3	66.4
5	FLAP 5	38	M	FAIS	3	71.4
6	FLAP 6	27	M	FAIS	3	28.5
7	FLAP 7	25	M	FAIS	3	77.2
8	FLAP 8	35	F	FAIS	3	124.3
9	FLAP 9	32	M	FAIS	3	38.2
10	FLAP 10	33	M	FAIS	3	195.6
11	FLAP 11	29	M	FAIS	3	34.0
12*^*b*^*	CR-a 1	54	M	AVN	—	116.6
12*^*b*^*	CR-a 2	54	M	AVN	—	71.2
13	CR 1	59	F	CL MFC	—	27.7
14	CR 2	43	F	CL MFC	—	76.6
15	CR 3	45	F	CL MFC	—	69.1
16	CR 4	20	M	CL RP	—	7.5
17	CR 5	51	F	CL MFC	—	136
18	CR 6	33	F	CL RP	—	61.3
19	CR 7	45	M	CL TRO	—	38.7
20	CR 8	28	M	CL MFC	—	180.9
21	CR 9	43	M	CL MFC	—	268.3
22	CR 10	25	M	CL MFC	—	73.5
23	CR 11	27	F	CL RP	—	17.6
24	CR 12	50	M	CL MFC	—	38.6

a ALAD, acetabular labrum articular disruption; AVN, avascular necrosis; CL, cartilage lesion; CR, control sample (from the knee); CR-a, control sample from the acetabulum; FAIS, femoroacetabular impingement syndrome; MFC, medial femoral condyle; RP, retro patella; TRO, trochlea.

b Cartilage biopsy from anatomically correlated regions of a patient during primary hip arthroplasty.

### Synchrotron Radiation Microcomputed Tomography Analyses

Seven FLAP samples were divided for imaging analyses and cell isolation. Only these 7 FLAP samples provided sufficient tissue volume to allow for both SR-μCT imaging and chondrocyte isolation and culture. The samples obtained for imaging were fixed in PBS (Bio&Sell GmbH) containing 4% v/v formaldehyde (Carl Roth) overnight and stored in PBS and fixed mechanically in 2-mL screw-cap tubes for subsequent SR-µCT analyses. These 7 FLAP samples and both CR-a samples were imaged using the SR-µCT setup at the ANATOMIX Beamline of the Synchrotron Facility (SOLEIL) in Paris, France, prior to histology.^32^ ANATOMIX is an Equipment of Excellence (EQUIPEX) funded by the Investments for the Future program of the French National Research Agency (ANR), project NanoimagesX, grant ANR-11-EQPX-0031. Samples were scanned entirely at an energy of 40 keV and an isotropic voxel size of 3.07 µm. To increase contrast in these low-absorbing samples, filtered back-projection, combined with a phase-retrieval step, was used to reconstruct the projection images. The segmentation of the cartilage cells and the surrounding tissue was done with a combination of gray-value filtering and morphologic operation steps. Segmentation and analysis were done using Thermo Scientific Avizo Software (Thermo Fisher Scientific) and MATLAB (The MathWorks).

### Histology

The histology of the fixed cartilage samples (from FLAP sample 7 and CR sample 4) and in vitro generated chondrogenic spheroids was performed as previously described in detail.^10,35^ In brief, the samples were dehydrated, embedded in paraffin, and sectioned using a microtome. The sections were rehydrated and stained with 1% Alcian blue 8GX solution (Morphisto) in 3% acetic acid (Th. Geyer) for 30 minutes and counterstained with nuclear fast red (Carl Roth) for 5 minutes. After dehydration and covering, imaging of the stained sections was performed with a hybrid microscope (Rebel; ECHO).

### Isolation, Cultivation, and Cryopreservation of Primary Human Chondrocytes

All of the FLAP and control samples (CR-a and CR) were weighed. FLAP and CR samples were cut into 1 × 1–mm pieces by a scalpel and were subsequently transferred to 6-well tissue culture plates at a weight to surface area ratio of 25 mg/10 cm^2^ and cultured in low-glucose Dulbecco's modified Eagle's medium (PAN Biotech) supplemented with 10% fetal bovine serum (Sigma-Aldrich), 100 U/mL penicillin (Gibco), 100 µg/mL streptomycin (Gibco), and 2 mM L-alanyl-L-glutamine (GlutaMAX; Gibco) at 37°C in a 5% CO_2_ atmosphere. Culture media were changed twice a week. Cells were detached on day 14 of primary cell culture by 0.05% trypsin containing 0.02% EDTA (PAN Biotech), and cell numbers were quantified using the TC20 Automated Cell Counter (Bio-Rad). Cells were seeded at a density of 1.0 × 10^3^ cells/cm^2^ and cultured until 80% confluence. The isolated chondrocytes were cryopreserved in cell culture passage 2 in low-glucose Dulbecco's modified Eagle's medium containing 12.5% human serum albumin (Biotests Pharma GmbH) and 10% dimethyl sulfoxide (AppliChem). The chondrocytes were thawed and further expanded for subsequent in vitro experiments.

### Cell Viability and Proliferation

The isolated chondrocytes were examined for their metabolic activity as a marker for cell viability and for their proliferation capacity in cell culture passage 3, as previously described in detail.^10,35^ In brief, metabolic activity was quantified at day 0 (24 hours after seeding), day 4, and day 7 by using the PrestoBlue assay (Invitrogen). Cell proliferation was quantified at day 4 and day 7 by using the CyQuant Cell Proliferation Assay (Thermo Fisher Scientific).

### Chondrogenic Redifferentiation

Chondrocytes from cell culture passage 4 were used to generate spheroids consisting of 3 × 10^5^ cells as previously described in detail.^10,35^ In brief, the spheroids were cultured in chondrogenic medium with or without the addition of recombinant human TGF-β1 (BioLegend) at a concentration of 10 ng/mL under hypoxic conditions to assess the specific influence of TGF-β1, a key growth factor present in platelet-rich plasma (PRP), as PRP-augmented minced cartilage procedures are increasingly discussed. After 21 days of 3D culture, 1 spheroid per group was fixed for histologic evaluation, and 3 spheroids per group were pooled for protein and proteoglycan quantification. After mechanically assisted chemical digestion of the spheroids, the total protein content was quantified by using the Bradford protein assay (Pierce Coomassie Protein Assay Kit; Thermo Fisher Scientific). The proteoglycan content was quantified as previously described in detail.^1^

### Statistical Analyses

Data plotting and statistical analyses were performed with GraphPad Prism (version 8.4.3; GraphPad Software) and MATLAB (The MathWorks). Detailed information about the statistical analyses of the individual experiments are included in the figure captions.

## Results

### Morphometric Evaluation

**Figure 1.**
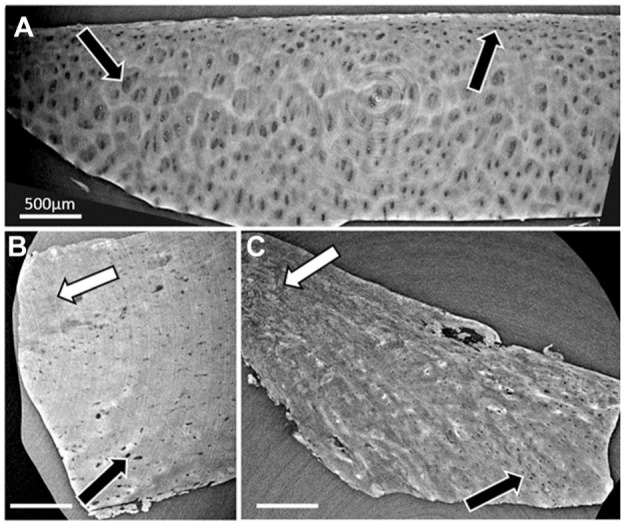
Representative computed tomography slices of (A) a control sample and (B, C) 2 morphologically distinctly different FLAP samples. Black arrows mark chondrocyte lacunae, and bright arrows mark cell-free areas with a fibrous appearance. In all images, the superficial cartilage surface is oriented toward the top.

SR-µCT images of 7 delaminated acetabular cartilage FLAP samples were compared with both CR-a samples. The analyses revealed a significantly altered structure of the cartilage tissue in FLAP compared with CR-a samples. In the control samples, chondrocyte lacunae of different sizes were clearly resolved and separated by denser borders (Figure 1A). All FLAP samples had a significantly altered appearance: the lacunae were smaller and varied in size, and the dense borders seemed to have vanished. Additionally, cell-free areas with a fibrous appearance were observed in the FLAP samples (Figure 1B).

**Figure 2.**
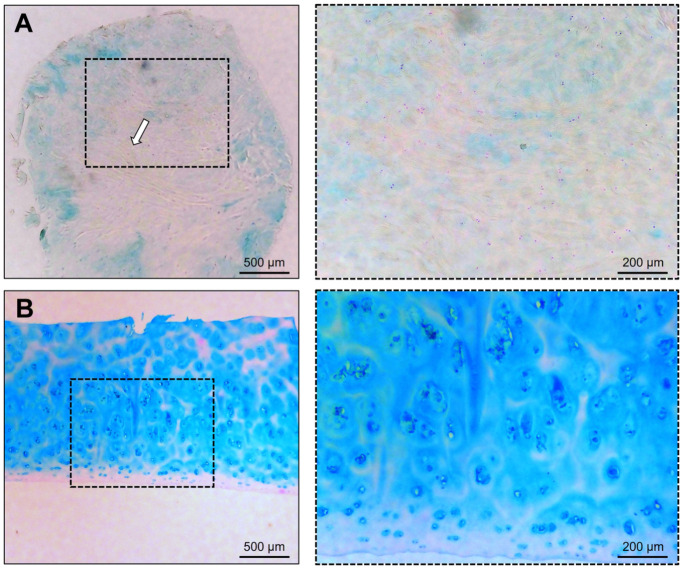
Histologic analyses show (A) cell-free, necrotic areas with fiber structures (white arrow) in a representative FLAP sample and (B) a reduced amount of glycosaminoglycans compared with a control sample.

Histologic evaluation confirmed focal tissue necrosis and the fibrocartilage-like tissue structure (Figure 2).

**Figure 3.**
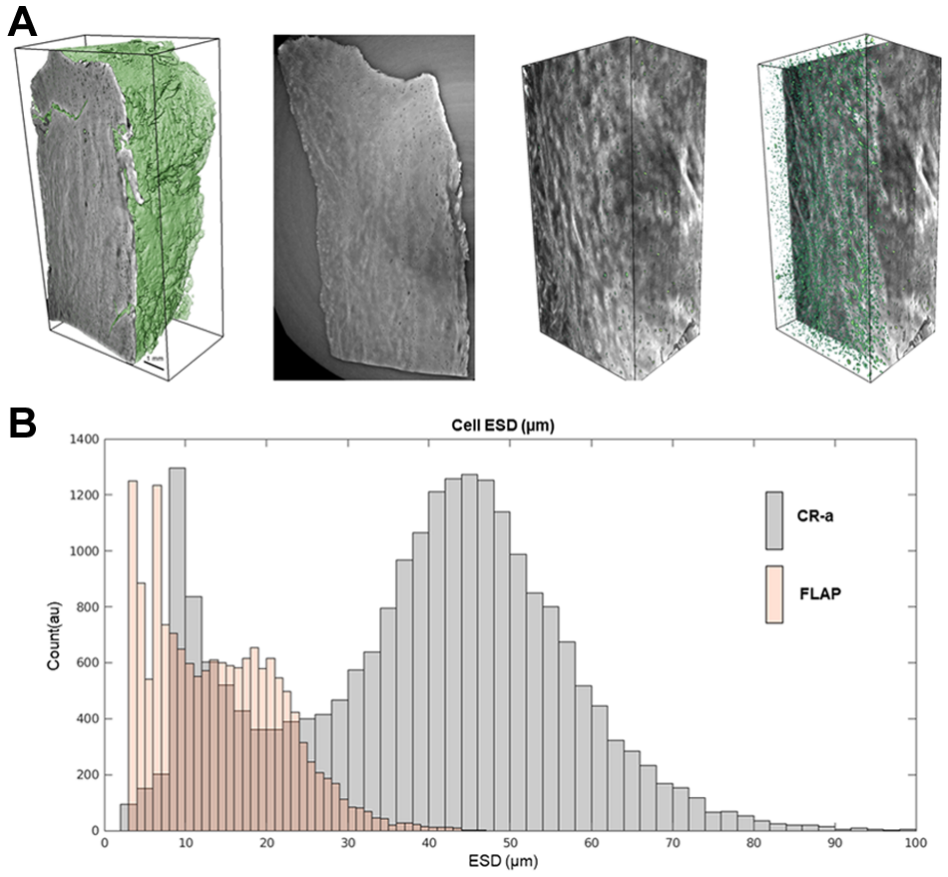
(A) Two-dimensional slice and 3-dimensional representation, including segmented chondrocyte lacunae (green) from FLAP sample computed tomography data. (B) Morphometric evaluation of the size and number of chondrocyte lacunae in comparison between control (CR-a) and FLAP (FLAP 7) samples. ESD, equivalent sphere diameter.

The quantitative analysis following segmentation of cell lacunae and plotting of the frequency distribution by lacuna size showed that, with a maximum diameter of 40 µm, the cell lacunae in the FLAP samples were smaller than the cell lacunae segmented in the control samples (Figure 3A). The control samples had a median lacuna diameter of 41.2 µm and 46.7 µm. In contrast, the segmented lacunae in the FLAP samples had a median diameter of 6 to 17 µm (Figure 3B, Table 2). Moreover, quantitative analysis of the number of chondrocyte lacunae per volume revealed a distinctively higher number of lacunae in FLAP samples (4372/mm^3^) compared to control samples (1158/mm^3^) (Table 2).

**Table 2:** Size and Number of Segmented Chondrocyte Lacunae*^*a*^*

Sample ID	Median Diameter, µm	Observed Volume, mm^3^	Detected Lacunae, No.	Lacunae/Volume, n/mm^3^
FLAP 1	10.7	3.1	11,205	3569
FLAP 2	6.0	2.7	14,299	5289
FLAP 3	13.1	23.0	102,532	4454
FLAP 7	13.2	2.2	15,740	7235
FLAP 8	12.2	1.0	4090	4107
FLAP 10	15.5	7.8	24,098	3079
FLAP 11	17.0	1.4	8165	5712
**Total FLAP**	**—**	**41.2**	**180,129**	**4372**
CR-a 1	41.2	17.9	22,562	1264
CR-a 2	46.7	8.9	8462	1010
**Total CR-a**	**—**	**26.8**	**31,024**	**1158**

a CR-a, control sample from the acetabulum.

### Comparison of Cell Outgrowth

**Figure 4.**
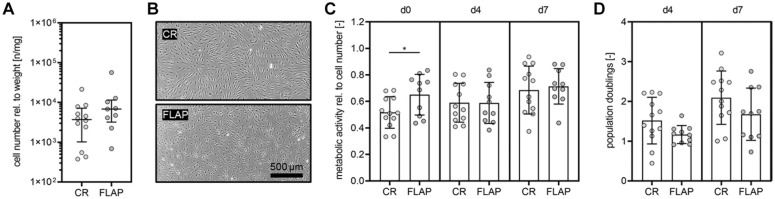
Cell yield, cell viability, and proliferation capacity. (A) Number of isolated chondrocytes at days 14 to 16 of primary cell culture normalized to the weight of seeded cartilage. (B) Representative phase contrast microscopy of chondrocytes in cell culture passage 2. (C) Metabolic activity as a marker for cell viability of chondrocytes at day 0 (24 hours after seeding), day 4, and day 7. (D) Cell population doublings of chondrocytes at day 4 and day 7 of cell culture (nonnormally distributed data: median with IQR, Mann-Whitney test; normally distributed data: mean ± SD, unpaired *t* test; levels of significance: *P < .05). CR, control; d0, day 0; d4, day 4; d7, day 7.

The number of outgrown chondrocytes was quantified after primary cell culture for 14 to 16 days, normalized to the seeded quantity of cartilage, and compared with the number of cells outgrown from control cartilage tissue taken from knee joints (CR). The analysis revealed no statistically significant difference in the median number of cells after in vitro outgrowth from isolated delaminated acetabular cartilage FLAP samples (6800 cells/mg; IQR, 3200-11,500) in comparison to CR samples (3715 cells/mg; IQR, 1018-7147; *P* = .19) (Figure 4, A and B).

### Comparison of Chondrocyte Viability and Proliferation Capacity

Metabolic activity and cell numbers were quantified to analyze whether chondrocytes isolated from delaminated acetabular cartilage FLAP samples differ with regard to cell viability and proliferation capacity from chondrocytes isolated from CR cartilage tissue. The metabolic activity at day 0, normalized to the corresponding cell number, was higher in chondrocytes isolated from delaminated acetabular cartilage FLAP samples (relative viability ± SD at day 0: 0.65 ± 0.15) in comparison to control cartilage samples (0.52 ± 0.12, *P* = .032). No difference between both groups was found at days 4 and 8 (Figure 4C). Furthermore, the proliferation capacity of chondrocytes isolated from delaminated acetabular cartilage FLAP samples was represented by the analysis of population doublings at day 4 (1.17 ± 0.23) and day 7 (1.68 ± 0.66) of cell culture and did not differ from CR samples at both time points (1.52 ± 0.59 and 2.09 ± 0.67, all *P* > .05) (Figure 4D).

### Comparison of Chondrogenic Redifferentiation Potential

**Figure 5.**
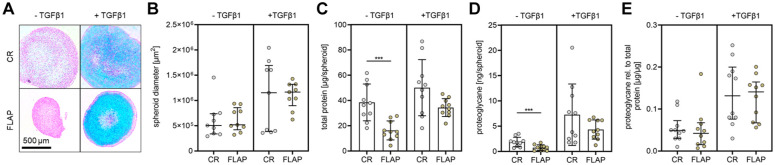
Chondrogenic redifferentiation after 21 days of 3-dimensional culture without chondrogenic stimulus (–TGFβ1) and with chondrogenic stimulus (+TGFβ1). (A) Representative images of alcian blue/nuclear fast red–stained sections of chondrogenic spheroids. (B) Quantification of the spheroids’ surface areas. (C) Total protein content of spheroids. (D) Total proteoglycan content of spheroids. (E) Total proteoglycan content normalized to total protein content of spheroids (nonnormally distributed data: median with IQR, Mann-Whitney test; normally distributed data: mean ± SD, unpaired *t* test; levels of significance: ***P < .001. CR, control.

Chondrocytes isolated from FLAP samples and chondrocytes isolated from CR samples were separately cultured in 3D spheroids. To analyze the potential of isolated cells from both groups for chondrogenic redifferentiation in 3D culture, respective subgroups were stimulated with TGF-β1. Following TGF-β1 stimulation, representative histologic sections of chondrocyte spheroids from delaminated acetabular cartilage FLAP and control cartilage samples accumulated glycosaminoglycans, as indicated by alcian blue staining (Figure 5A). The addition of TGF-β1 to chondrocyte 3D culture increased the diameter of chondrocyte spheroids in general. However, no differences were observed in the diameter of 3D spheroids cultured from chondrocytes derived from delaminated acetabular cartilage FLAP and CR samples (Figure 5B). If cultured without TGF-β1, the total protein content per spheroid was significantly reduced in the delaminated acetabular cartilage FLAP group (16.25 ± 7.52 µg/spheroid) compared with the CR group (38.46 ± 14.57 µg/spheroid, *P* < .001). Following stimulation of 3D spheroids with TGF-β1, no difference in total protein content was observed between the groups (34.36 ± 7.07 µg/spheroid vs 50.15 ± 22.23 µg/spheroid, *P* = .056) (Figure 5C). In absence of TGF-β1, the proteoglycan content per spheroid was significantly reduced in the delaminated acetabular cartilage FLAP group (0.74 ± 0.57 ng/spheroid) compared with the CR group (1.86 ± 0.93 ng/spheroid, *P* < .01), and no difference in proteoglycan content was observed if TGF-β1 was added (4.34 ± 1.9 ng/spheroid vs 7.27 ± 6.09 ng/spheroid, *P* = .17) (Figure 5D). Stimulation with TGF-β1 led to an increase in proteoglycan concentration normalized to total protein content in both groups, while no differences between the groups were observed (Figure 5E).

## Discussion

The main findings of our study are that, according to SR-µCT imaging, the tissue structure of delaminated acetabular cartilage FLAP samples is severely altered, showing smaller and more chondrocyte lacunae compared to intact acetabular cartilage. Furthermore, in FLAP samples, cell-free areas with fibrous appearance were observed by SR-µCT and histology. No differences regarding outgrowth potential, cell viability, and population doublings of chondrocytes isolated from FLAP samples in comparison to chondrocytes derived from knee joints during MCI were observed. Chondrogenic redifferentiation and extracellular matrix production of chondrocytes isolated from delaminated acetabular cartilage FLAP samples required the addition of TGF-β1. The results indicate that delaminated acetabular cartilage could be an adequate tissue source for MCI, while augmentation with TGF-β1 containing orthobiologics seems to be beneficial.

Earlier studies on the morphologic structure and composition of delaminated acetabular cartilage tissue revealed that only 50% of investigated samples were composed of hyaline-like cartilage,^22^ collagen content and chondrocyte biosynthesis was reduced,^14^ degenerative changes were histologically evident,^24^ and disrupted extracellular matrix and isolated chondrocytes showed a qualitatively lower chondrogenic potential with inhomogeneous cell and matrix distribution compared to controls.^19^ In the present study, for the first time, delaminated acetabular cartilage FLAP samples were investigated in 3D by SR-µCT. SR-µCT provides high-resolution, nondestructive 3D imaging of mineralized and soft tissues, with voxel sizes reaching below 1 µm.^16^ In contrast to conventional absorption-based µCT, SR-µCT enables phase-contrast imaging, which significantly enhances the visualization of low-density tissues such as cartilage, especially at the cellular and pericellular level.^34^ In cartilage analysis, SR-µCT has been successfully used to visualize and quantify chondrocyte lacunae morphology and density, detect early signs of matrix degeneration, and distinguish spatial alterations in lacunar organization between healthy and osteoarthritic tissue.^7,15^ Recent studies demonstrated that SR-µCT can assess cartilage matrix integrity and cellular organization in unprecedented detail, offering a volumetric alternative to classical histology.^16^ Especially evident in our investigation were alterations of chondrocyte lacunae with a decrease in lacunae size and a significant increase in lacunae numbers per area in delaminated acetabular chondral flap tissue compared to cartilage tissue derived from an intact acetabulum during THA for avascular necrosis. In this regard, it remains unclear whether the smaller lacunae with borders of lesser density in FLAP samples were probably remnants of former bigger lacunae, as observed in control samples.

Despite the unequivocal finding of profound morphometric discrepancies between the structure of delaminated acetabular cartilage tissue and healthy hyaline-like cartilage in the literature and the present study, earlier clinical studies reported pain relief and improved function following reattachment of delaminated acetabular chondral flaps by fibrin glue^30,31^ or suture repair^28^ at short-term follow-up. However, a recent systematic review analyzing the long-term results of direct repair of delaminated acetabular chondral flaps found the conversion rate to THA equally high compared with debridement and consecutive MFX, a technique that has been abandoned by some surgeons.^21^ In the same review article, reduced conversion rates to THA following treatment with autologous matrix-induced chondrogenesis (AMIC) compared to MFX were reported. Accordingly, in a comparative study, lower conversion rates to THA and better mid-term functional clinical outcomes for AMIC compared to MFX were reported.^11^ While the comparative study by Girolamo et al^11^ reported significantly better functional outcome for AMIC compared to MFX, the absolute outcome of the AMIC group was reported with a moderate modified Harris Hip Score of around 80 points. In comparison, recently reported outcomes for ACI treatment (mean modified Harris Hip Score of 92.2 ± 7.2)^18^ and MCI (International Hip Outcome Tool 12 score of 86.5 ± 19)^9^ were higher. Although such a comparison between different studies is of limited significance, it may serve as a first reference that cell-based cartilage repair, especially the integrity of the subchondral bone, may be favorable in the treatment of acetabular cartilage lesions.

While to date, the best evidence is provided for the ACI, this technique has distinct disadvantages in everyday practice, as it requires 2 operative procedures, is relatively expensive, and is subject to regulatory provisions.^2^ Therefore, the MCI could be the ideal technique to treat acetabular cartilage lesions, since it is cell based, provides integrity of the subchondral bone, can be performed in a single operation, and uses tissue from the available delaminated acetabular cartilage flap.

While early clinical results for MCI at the hip are encouraging,^9^ controlled clinical studies of high evidence level are missing to date. Furthermore, in vitro research on MCI has shown that chondrocyte outgrowth and functionality rely on the donor cartilage tissue.^10^ Thus, it has to be clarified whether tissue derived from delaminated acetabular cartilage is a suitable source for MCI. Interestingly, despite the significant morphologic differences observed between delaminated acetabular cartilage and intact control cartilage, the results of the present study indicate that chondrocytes isolated from delaminated tissue retained a comparable capacity for proliferation and viability when cultured under standard conditions, including in the presence of TGF-β1. Clinically, this is of particular relevance because TGF-β1 is a key component of PRP, which is widely used to biologically augment MCI procedures, ensuring that transplanted chondrocytes are typically exposed to TGF-β1 in vivo. This finding is indeed unexpected and underscores the potential of even structurally compromised cartilage to serve as a viable cell source for regenerative purposes. Although we did not directly quantify apoptosis rates or metabolic activity in this study, the robust proliferation observed suggests that a considerable proportion of chondrocytes from delaminated tissue remains functionally intact. Future studies incorporating specific viability assays and apoptosis markers would be valuable to further elucidate this observation. Furthermore, our results are in line with earlier findings showing that the viability of chondrocytes isolated from delaminated acetabular cartilage tissue is equal to that of chondrocytes isolated from cartilage of the knee.^5^ However, the present study additionally revealed that the proteoglycan content, a prerequisite for chondrogenic extracellular matrix production, of chondrocytes derived from delaminated acetabular cartilage was significantly lower compared with cartilage tissue from the knee joint. Relevant proteoglycan synthesis in chondrocytes derived from delaminated acetabular cartilage samples was only observed following stimulation with TGF-β1, which is known to induce proteoglycan and collagen type II synthesis of chondrocytes in vitro.^13,17,25^ Accordingly, our research group recently reported a positive effect of PRP, an orthobiologic containing TGF-β1, on proteoglycan production of chondrocytes in conjunction with MCI in cartilage tissue taken from the knee joint.^10^ Taken together, our in vitro analyses indicate that tissue from delaminated acetabular cartilage augmented with PRP seems to be an adequate source for MCI at the hip joint.

### Limitations

A limitation of this study is that the cartilage used for comparison was derived from different patients and from knee joints; thus, no statement can be made whether cartilage from other areas of the hip joint would be a better source for MCI, and undesirable influences cannot be excluded. Furthermore, the knee cartilage specimens originated from areas adjacent to traumatically altered or overloaded regions and may therefore not represent completely “normal” reference tissue. The analysis of SR-µCT imaging was challenging due to the significantly altered structure of the samples and the fact that only soft tissue was examined, making it difficult to distinguish structures and boundaries. The differences in lacuna size and number are distinct. However, the stated precise measurements of the size and number of lacunae should be interpreted with caution. The study investigated chondrocyte viability and functionality of delaminated acetabular cartilage and found that they might be suitable for MCI, but the results cannot be used to predict the quality and durability of the regenerated cartilage and long-term results of MCI at the hip joint. Thus, this study cannot replace controlled clinical studies on MCI for the treatment of delaminated acetabular cartilage lesions.

## Conclusion

Delaminated acetabular cartilage has a significantly altered morphologic structure. Cell numbers, outgrowth potential, and population doublings of chondrocytes isolated from delaminated acetabular cartilage were comparable to control cartilage samples, indicating their suitability to serve as a tissue source for MCI. However, the ability of chondrocytes isolated from delaminated acetabular cartilage to produce proteoglycans was dependent on stimulation by TGF-β1. Thus, MCI treatment at the hip might benefit from augmentation with TGF-β1.

## References

[1] Andrzejewska A Catar R Schoon J , et al. Multi-parameter analysis of biobanked human bone marrow stromal cells shows little influence for donor age and mild comorbidities on phenotypic and functional properties. Front Immunol. 2019;10:2474. 31781089 10.3389/fimmu.2019.02474 PMC6857652

[2] Armoiry X Cummins E Connock M , et al. Autologous chondrocyte implantation with chondrosphere for treating articular cartilage defects in the knee: an evidence review group perspective of a NICE single technology appraisal. PharmacoEconomics. 2019;37(7):879-886. 30426462 10.1007/s40273-018-0737-z

[3] Barbaret A Wein F Jacquet C Ollivier M. One-stage minced cartilage autograft with platelet-rich plasma improves early clinical outcomes: a multicentric retrospective study. J Exp Orthop. 2025;12(1):e70162. 10.1002/jeo2.70162 PMC11808269 39931147

[4] Blanke F Warth F Oehler N Siegl J Prall WC. Autologous platelet-rich plasma and fibrin-augmented minced cartilage implantation in chondral lesions of the knee leads to good clinical and radiological outcomes after more than 12 months: a retrospective cohort study of 71 patients. J Exp Orthop. 2024;11(4):e70051. 10.1002/jeo2.70051 PMC11480521 39415804

[5] Bretschneider H Stiehler M Hartmann A , et al. Characterization of primary chondrocytes harvested from hips with femoroacetabular impingement. Osteoarthritis Cartilage. 2016;24(9):1622-1628. 27084349 10.1016/j.joca.2016.04.011

[6] Clohisy JC Baca G Beaulé PE , et al. Descriptive epidemiology of femoroacetabular impingement: a North American cohort of patients undergoing surgery. Am J Sports Med. 2013;41(6):1348-1356. 23669751 10.1177/0363546513488861

[7] Danalache M Beutler KR Rolauffs B , et al. Exploration of changes in spatial chondrocyte organisation in human osteoarthritic cartilage by means of 3D imaging. Sci Rep. 2021;11(1):9783. 33963289 10.1038/s41598-021-89582-w PMC8105369

[8] El Bitar YF Lindner D Jackson TJ Domb BG. Joint-preserving surgical options for management of chondral injuries of the hip. J Am Acad Orthop Surg. 2014;22(1):46-56. 24382879 10.5435/JAAOS-22-01-46

[9] Gebhardt S Hofer A Wassilew GI Sobau C Zimmerer A. Minced cartilage implantation in acetabular cartilage defects: case series with 2-year results. Cartilage. 2023;14(4):393-399. 37533396 10.1177/19476035231189840 PMC10807734

[10] Gebhardt S Zimmerer A Balcarek P Wassilew GI Schoon J. The influence of arthroscopic shaver mincing and platelet-rich plasma on chondrocytes of intraoperatively harvested human cartilage. Am J Sports Med. 2023;51(10):2679-2687. 37449659 10.1177/03635465231181633 PMC10394959

[11] Girolamo L de Jannelli E Fioruzzi A Fontana A. Acetabular chondral lesions associated with femoroacetabular impingement treated by autologous matrix-induced chondrogenesis or microfracture: a comparative study at 8-year follow-up. Arthroscopy. 2018;34(11):3012-3023. 30266548 10.1016/j.arthro.2018.05.035

[12] Green CJ Beck A Wood D Zheng MH. The biology and clinical evidence of microfracture in hip preservation surgery. J Hip Preserv Surg. 2016;3(2):108-123. 27583147 10.1093/jhps/hnw007 PMC5005050

[13] Grimaud E Heymann D Rédini F. Recent advances in TGF-beta effects on chondrocyte metabolism. Potential therapeutic roles of TGF-beta in cartilage disorders. Cytokine Growth Factor Rev. 2002;13(3):241-257. 12486877 10.1016/s1359-6101(02)00004-7

[14] Hariri S Truntzer J Smith RL Safran MR. Biochemical and cellular assessment of acetabular chondral flaps identified during hip arthroscopy. Arthroscopy. 2015;31(6):1077-1083. 25749531 10.1016/j.arthro.2015.01.010

[15] Honkanen MKM Saukko AEA Turunen MJ , et al. Synchrotron MicroCT reveals the potential of the dual contrast technique for quantitative assessment of human articular cartilage composition. J Orthop Res. 2020;38(3):563-573. 31535728 10.1002/jor.24479 PMC7065106

[16] Horng A Stroebel J Geith T , et al. Multiscale X-ray phase contrast imaging of human cartilage for investigating osteoarthritis formation. J Biomed Sci. 2021;28(1):42. 34098949 10.1186/s12929-021-00739-1 PMC8182937

[17] Kabiri A Esfandiari E Esmaeili A Hashemibeni B Pourazar A Mardani M. Platelet-rich plasma application in chondrogenesis. Adv Biomed Res. 2014;3:138. 25161985 10.4103/2277-9175.135156 PMC4139981

[18] Krueger DR Baur ADJ Perka C Schroeder JH. Injectable autologous chondrocyte implantation in acetabular cartilage defects: 2-year minimum clinical and MRI results. Arch Orthop Trauma Surg. 2023;143(2):739-747. 34468836 10.1007/s00402-021-04141-2

[19] Levinson C Naal FD Salzmann GM Zenobi-Wong M Leunig M. Is there a scientific rationale for the refixation of delaminated chondral flaps in femoroacetabular impingement? A laboratory study. Clin Orthop Relat Res. 2020;478(4):854-867. 32011382 10.1097/CORR.0000000000001135 PMC7282577

[20] Lodhia P Gui C Chandrasekaran S Suarez-Ahedo C Vemula SP Domb BG. Microfracture in the hip: a matched-control study with average 3-year follow-up. J Hip Preserv Surg. 2015;2(4):417-427. 27011867 10.1093/jhps/hnv073 PMC4732373

[21] Lu V Andronic O Zhang JZ Khanduja V. Outcomes of arthroscopy of the hip for femoroacetabular impingement based on intraoperative assessment using the Outerbridge classification. Bone Joint J. 2023;105-B(7):751-759. 37399116 10.1302/0301-620X.105B7.BJJ-2022-0989.R1

[22] Meulenkamp B Gravel D Beaulé PE. Viability assessment of the chondral flap in patients with cam-type femoroacetabular impingement: a preliminary report. Can J Surg. 2014;57(1):44-48. 24461226 10.1503/cjs.003513 PMC3908995

[23] Pascual-Garrido C Li DJ Grammatopoulos G Yanik EL Clohisy JC. The pattern of acetabular cartilage wear is hip morphology-dependent and patient demographic-dependent. Clin Orthop Relat Res. 2019;477(5):1021-1033. 30998630 10.1097/CORR.0000000000000649 PMC6494325

[24] Rodriguez-Fontan F Payne KA Chahla J , et al. Viability and tissue quality of cartilage flaps from patients with femoroacetabular hip impingement: a matched-control comparison. Orthop J Sports Med. 2017;5(8):2325967117723608. 10.1177/2325967117723608 PMC5562333 28868322

[25] Rosadi I Karina K Rosliana I , et al. In vitro study of cartilage tissue engineering using human adipose-derived stem cells induced by platelet-rich plasma and cultured on silk fibroin scaffold. Stem Cell Res Ther. 2019;10(1):369. 31801639 10.1186/s13287-019-1443-2 PMC6894137

[26] Runer A Ossendorff R Öttl F , et al. Autologous minced cartilage repair for chondral and osteochondral lesions of the knee joint demonstrates good postoperative outcomes and low reoperation rates at minimum five-year follow-up. Knee Surg Sports Traumatol Arthrosc. 2023;31(11):4977-4987. 37634136 10.1007/s00167-023-07546-1 PMC10598129

[27] Schneider S Ossendorff R Walter SG , et al. Arthroscopic autologous minced cartilage implantation of cartilage defects in the knee: a 2-year follow-up of 62 patients. Orthop J Sports Med. 2024;12(12):23259671241297970. 10.1177/23259671241297970 PMC11618912 39640183

[28] Sekiya JK Martin RL Lesniak BP. Arthroscopic repair of delaminated acetabular articular cartilage in femoroacetabular impingement. Orthopedics. 2009;32(9):102950. 10.3928/01477447-20090728-44 19750994

[29] Sogbein OA Shah A Kay J , et al. Predictors of outcomes after hip arthroscopic surgery for femoroacetabular impingement: a systematic review. Orthop J Sports Med. 2019;7(6):2325967119848982. 10.1177/2325967119848982 PMC6585257 31259183

[30] Stafford GH Bunn JR Villar RN. Arthroscopic repair of delaminated acetabular articular cartilage using fibrin adhesive. Results at one to three years. Hip Int. 2011;21(6):744-750. 22117261 10.5301/HIP.2011.8843

[31] Tzaveas AP Villar RN. Arthroscopic repair of acetabular chondral delamination with fibrin adhesive. Hip Int. 2010;20(1):115-119. 20235074 10.1177/112070001002000117

[32] Weitkamp T Scheel M Perrin J , et al. Microtomography on the ANATOMIX beamline at Synchrotron SOLEIL. J Phys Conf Ser. 2022;2380(1):12122.

[33] Wright VJ McCrum CL Li H Tranovich MJ Huard J. Significant chondrocyte viability is present in acetabular chondral flaps associated with femoroacetabular impingement. Am J Sports Med. 2018;46(1):149-152. 29024597 10.1177/0363546517732751

[34] Zehbe R Riesemeier H Kirkpatrick CJ Brochhausen C. Imaging of articular cartilage—data matching using X-ray tomography, SEM, FIB slicing and conventional histology. Micron (Oxford, England : 1993). 2012;43(10):1060-1067. 22633854 10.1016/j.micron.2012.05.001

[35] Zimmerer A Schulze F Gebhardt S , et al. Impact of gadolinium-based MRI contrast agent and local anesthetics co-administration on chondrogenic gadolinium uptake and cytotoxicity. Heliyon. 2024;10(8):e29719. 10.1016/j.heliyon.2024.e29719 PMC11053198 38681575

